# Smart@home – supporting safety and mobility of elderly and care dependent people in their own homes through the use of technical assistance systems and conventional mobility supporting tools: a cross-sectional survey

**DOI:** 10.1186/s12877-021-02118-9

**Published:** 2021-03-24

**Authors:** Deborah Elisabeth Jachan, Ursula Müller-Werdan, Nils Axel Lahmann, Sandra Strube-Lahmann

**Affiliations:** 1 Department of Geriatrics, Charité – Universitätsmedizin Berlin, corporate member of Freie Universität Berlin, Humboldt-Universität zu Berlin, and Berlin Institute of Health, Augustenburger Platz 1, 13353 Berlin, Germany; 2 Department of Geriatrics, Geriatrics Research Group, Charité – Universitätsmedizin Berlin, Reinickendorfer Straße 61, 13347 Berlin, Germany

**Keywords:** Communication, Mobility, Quality of life, Safety, Smart home, Usability, User satisfaction

## Abstract

**Background:**

The use of technical solutions and conventional mobility supporting aids can support the independence of people into old age in their own homes. However, we found relatively few empirical investigations on the effects and costs of these systems.

**Methods:**

The aim of the study was to investigate usability, user satisfaction and the correlation between costs and benefits of different built-in smart home solutions and conventional mobility supporting tools in the home of elderly, partially care-dependent tenants (> 65 years). A cross-sectional survey was conducted from February to March 2018 with tenants of a housing association in apartments equipped with smart home technology and conventional mobility supporting tools. The response rate in the intervention group was *n* = 37 persons (out of 46 tenants with installed smart home and conventional solutions) and in the control group *n* = 64 persons (out of 100 tenants without built-in smart home and conventional solutions). Data were collected by a written questionnaire regarding usability and satisfaction of the tenants with the built-in smart home solutions and conventional mobility supporting tools. In addition, both the intervention and the control group were asked general questions about communication, safety and how to deal with the need for long-term care in their own living environment.

**Results:**

Results showed that with regard to usability, satisfaction and price performance ratio of the installed smart home solutions, the installation of the corresponding solutions with an overall score of 1.41 (on a scale of 1 (very good) to 6 (unsatisfactory)) was mostly positively evaluated by the tenants. Overall, users rated the installed smart home solutions better than the conventional mobility supporting tools (such as handholds and increased balcony floor level).

**Conclusions:**

Analysis of the price performance ratio showed that smart home solutions are generally more expensive than conventional tools, but also contribute significantly to an increased security of the tenants, and thus may enable longer living in a familiar environment. We recommend modularized offers consisting of various components of smart home solutions, since this significantly reduces installation costs and allows for an individual composition according to requirements. Moreover, smart home solutions should be considered to be listed as medical aids.

**Supplementary Information:**

The online version contains supplementary material available at 10.1186/s12877-021-02118-9.

## Background

Information and communication technologies are becoming increasingly important in the care of the elderly and persons in need of care [1, 2]. These technologies include the so-called *smart home*, which by definition is an information and sensor technology upgraded home that is networked within itself and externally [3]. Certain technical solutions can promote or maintain the independence of people into old age [4, 5]. In the meantime, more and more technical solutions are available for easy-to-use, barrier-free and age-appropriate care at home [6–8]. The need for such applications becomes essential in particular due to socio-demographic changes, the lack of skilled nursing staff, an increase in single and childless households as well as increasing mobility and growing distances between (care-receiving) parents and adult children. These changes in population and care structure are creating new demands and specific challenges for the entire health care system [9, 10].

The expected increase in the number of people in need of long-term care from 2.5 million in 2013 to up to 3.5 million in 2030 [11] is also expected to result in a growing proportion of old and very old people living in their own homes and/or receiving care there. Currently, about three quarters of all care recipients in Germany are cared for at home [12]. This corresponds to the desire of most people in need of long-term care to be cared for in their own homes [13] and also follows the guiding principle of the reform of the social long-term care insurance of “outpatient before inpatient” [14, 15].

With regard to the outpatient sector, intelligent living environments (smart home solutions) can provide important and necessary support for people in need of help and/or care in coping with their everyday life, maintaining their state of health and autonomy, social participation and increasing their security. In order to meet the desire of most people to be able to live as long and safely as possible in their own homes [16, 17], technical solutions and aids must meet special requirements [18]. They should be reliable, user-friendly, suitable for everyday use and robust, and also provide various expandable functions, such as fall detection sensors with integrated emergency call function and intelligent light strips for a safe orientation at night [19].

Nevertheless, besides the new smart home solutions, conventional mobility supporting tools, meaning non-technical/non-sensor-based aids, such as grab handles, service sockets and balcony elevations for easy entry and exit are well-known aids, which have been commonly installed and are still being used in apartments for older adults.

The present study was conducted to determine the extent to which smart home solutions and conventional mobility supporting tools can contribute to promoting and maintaining the independence of older adults and to increase safety in their own homes. Therefore, residents from a housing facility living in apartments with and without installed smart home solutions and conventional mobility supporting aids participated in the study, answered questions in a questionnaire, and finally returned them anonymously to the housing facility (data collection location). The following research questions were addressed in the investigation:

1. How do residents evaluate the effective use of smart home solutions and conventional mobility supporting tools in their own homes?
2. What is the price and quality performance ratio of the smart home solutions and conventional mobility supporting tools, taking into account the resulting benefit (efficiency) for the tenants?

The first research question addressed older adults’ general perceptions on how secure they feel in their own apartment and how smart home solutions and conventional mobility supporting tools may add to a secure living environment. In addition, residents from the intervention group were asked how they evaluated the installed smart home solutions and conventional mobility supporting tools. The Null hypothesis states that there is no difference between the intervention and control group. This approach allowed us to get a full picture on older adults’ general security perceptions with regard to smart home solutions and conventional mobility supporting aids to be possibly installed in future in combination with an evaluation of actually installed solutions. The comparison between smart (technically based) solutions and conventional (non-technical) mobility supporting aids was drawn in order to investigate which kind of solutions (technical or non-technical – or maybe a combination of both) support tenants’ needs for a secure living in the best possible way.

Based on the answers of the intervention group on quality performance, the second research question combined quality and price performance in order to find out, if highest prices correspond to best quality, and if best rated solutions are technical (smart home solutions) or non-technical (conventional mobility supporting tools).

## Method

### Study design

The present study was conducted from February to March 2018 in a district with an above-average number of elderly people. As part of a cross-sectional survey, tenants at the age of 65 to 90+, some of them living independently, others in need of care (Table 1), were asked to fill in a questionnaire (Supplementary file 1). In their apartments, individual smart home solutions and other conventional mobility supporting aids such as grab handles, service sockets and balcony elevations for easy entry and exit were previously installed between one and six months before study participation. The survey focused primarily on the aspects of the *need for care, communication/social contacts* and *security* (Table 2)*.* The questionnaire was pre-tested for comprehensibility and legibility by researchers and nurses in Geriatrics, and modified accordingly.

**Table 1:** Sample characteristics (n = 101)

	Intervention group in % (n)	Control group in % (n)
**Age**	**n = 35**	**n** **= 63**
65–70 years	20.0 (7)	20.6 (13)
71–75 years	17.1 (6)	19.0 (12)
76–80 years	37.1 (13)	19.0 (12)
81–85 years	20.0 (7)	25.4 (16)
86–90 years	5.7 (2)	14.3 (9)
> 90 years	0.0 (0)	1.6 (1)
**Sex**	**n = 37**	**n = 61**
Female	75.7 (28)	78.7 (48)
Male	24.3 (9)	21.3 (13)
**Need for care**	**n** **= 32**	**n** **= 59**
In need of care	43.80 (14)	13.60 (8)
Independent	56.30 (18)	86.40 (51)
**Care degrees**	**n** **= 15**	**n** **= 8**
Care degree 1	33.3 (5)	37.5 (3)
Care degree 2	33.3 (5)	50.0 (4)
Care degree 3	13.3 (2)	12.5 (1)
Care degree 4	20.0 (3)	0.0 (0)
**Physical limitations**	**n = 37**	**n = 64**
Chronic pain	48.60 (18)	40.60 (26)
Impaired mobility	59.50 (22)	43.80 (28)
Urinary incontinence	35.10 (13)	14.10 (9)
Other physical disorders	29.70 (11)	23.40 (15)

**Table 2:** Comparison of the living situation with and without smart home solutions (n = 101)

Statements on housing situation:	n	Mean	SD	1 fully agree	2 rather agree	3 partly agree	4 rather not agree	5 not agree	Chi^**2**^
				%					p
*Need for care*									
**I would like to live in my apartment even if the need for care arises.**	^a^36	1.31	0.75	83.3	5.6	8.3	2.8	0.0	0.09
	^b^64	1.44	0.69	67.2	21.9	10.9	0.0	0.0	
**Even with increasing need for care (up to being bedridden) I want to live in my apartment.**	37	1.89	1.24	59.5	8.1	21.6	5.4	5.4	0.22
	62	2.15	1.13	38.7	22.6	27.4	8.1	3.2	
**Smart home solutions (e.g. orientation light on the ground. Fall detection sensor) enable me to live longer in my own home.**	33	1.97	1.38	54.5	21.2	9.1	3.0	12.1	0.70
	53	2.15	1.31	39.6	32.1	13.2	3.8	11.3	
*Communication/social contacts*									
**I have a person with whom I am in regular contact to report that I am doing well.**	37	1.08	0.36	94.6	2.7	2.7	0.0	0.0	0.06
	63	1.70	1.27	69.8	11.1	6.3	4.8	7.9	
**I know whom I can inform immediately in the case of an emergency.**	35	1.09	0.28	91.4	8.6	0.0	0.0	0.0	0.23
	61	1.34	0.79	80.3	8.2	9.8	0.0	1.6	
**I leave my apartment regularly to maintain social contacts.**	36	1.50	1.00	75.0	8.3	11.1	2.8	2.8	0.23
	61	1.74	0.98	57.4	18.0	18.0	6.6	0.0	
*Safety*									
**I feel safe in my apartment.**	37	1.22	0.53	83.8	10.8	5.4	0.0	0.0	0.71
	61	1.30	0.59	77.0	16.4	6.6	0.0	0.0	
**The installed smart home solutions make me feel safer in my home.**	31	1.55	0.81	61.3	25.8	9.7	3.2	0.0	0.04
	26	2.38	1.39	34.6	23.1	26.9	0.0	15.4	
**Smart home solutions in my home lead to more security.**	31	1.71	0.97	54.8	29.0	6.5	9.7	0.0	0.16
	49	2.02	0.99	36.7	32.7	24.5	4.1	2.0	
**I can move independently in my apartment.**	37	1.38	0.79	78.4	8.1.	10.8	2.7	0.0	0.38
	63	1.48	0.78	69.8	12.7	17.5	0.0	0.0	
**I can orientate myself well in my apartment even in the dark.**	36	1.61	1.10	69.4	13.9	5.6	8.3	2.8	0.05
	63	1.79	0.94	52.4	19.0	25.4	3.2	0.0	
**I am afraid of forgetting to switch off electronic devices and causing fire damage.**	33	3.58	1.30	9.1	15.2	12.1	36.4	27.3	0.09
	63	3.30	1.32	9.5	19.0	30.2	14.3	27.0	
**I am afraid of forgetting to switch off electronic devices and causing water damage.**	33	3.73	1.16	6.1	9.1	18.2	39.4	27.3	0.29
	61	3.33	1.29	8.2	21,3	24.6	21.3	24.6	
**I feel comfortable in my apartment.**	37	1.24	0.76	86.5	8.1	2.7	0.0	2.7	0.73
	64	1.31	0.75	81.3	9.4	7.8	0.0	1.6	

^a^Intervention group with smart home solutions

^b^Control group without smart home solutions

### Sample

The written survey was conducted by means of questionnaires with an enclosed stamped envelope. The survey included all tenants aged 65 to 90+ years of a municipal social housing association, in 27 of whose apartments smart home solutions and/or conventional mobility supporting aids to support mobility had been installed. The apartments are located in a large city near the city center and are available for socially disadvantaged persons with lower incomes. As a control group, a random selection of 100 additional tenants with the same age structure was available, in whose apartments no smart home solutions or conventional mobility aids had been installed so far. In order to ensure the highest possible response rate, the tenants had the opportunity to complete the questionnaire at home. The tenants could either return the completed questionnaire directly by mail in a stamped envelope or in a sealed envelope to the housing association.

### Data collection

Tenants with installed smart home and conventional solutions were provided with information on the study and the course of the investigation at an information event. Afterwards they had the opportunity to fill in the 2-page questionnaire (Supplementary file 1) directly on site or to take it home and return it afterwards. Tenants of the control group received a letter from the research group by internal mail from their housing association with explanations of the survey and a 1-page questionnaire with a stamped envelope. The data collection was carried out according to the medical-ethical principles of the Ethics Committee of the State of Berlin.

The questionnaire was developed by the authors based on literature review and usability criteria developed in further research projects on care-dependent geriatric residents and patients with the use of new technologies and consisted of three parts in total. In the *first part,* demographic data was collected. In the *second part* of the questionnaire, the participants were asked to give an assessment of various aspects of their individual housing situation in order to be able to compare the intervention and control group with regard to *need for care, communication/social contacts* and *security* in combination with smart home solutions (Table 2). The respondents were able to indicate whether and to what extent the statements on the questionnaire were applicable using a 5-level scale (1 = “fully agree”; 2 = “rather agree”; 3 = “partly agree”; 4 = “rather not agree”; 5 = “not agree”). In the *third part* of the questionnaire, tenants with built-in solutions were asked about their satisfaction with the smart home solutions which had been installed as well as other built-in conventional mobility supporting aids (Table 3 offers a complete list of sensors and aids installed). In order to assess user satisfaction with the installed solutions, the participants in the intervention group were given a scale of 1 (very good) to 6 (unsatisfactory).

**Table 3:** Tenants’ satisfaction with installed smart home solutions and conventional mobility support aids (n = 37)

Installed smart home solutions:	Evaluation of tenants’ satisfaction on a scale from 1 (very good) to 6 (unsatisfactory) in %						n	Mean
	1	2	3	4	5	6		
**Tablet** Used to operate lighting control.	50.0	33.3	11.1	0	0	5.6	18	1.83
**Stove safety** The sensor detects whether the stove is in operation and whether a person is standing in front of it. If no movement is detected for a longer period, the stove will automatically be deactivated.	78.9	21.1	0	0	0	0	19	1.21
**Orientation light** The sensor activates glare-free floor lighting all the way into the bathroom and the ceiling light in the bathroom as soon as a person leaves the bed.	85.0	15.0	0	0	0	0	20	**1.15**
**Lighting control** Comfortable control of lamps via app on the tablet.	53.8	38.5	7.7	0	0	0	13	1.54
**LED strip (corridor)** Illuminates the corridor dimmable and glare-free at night on detected movement.	66.7	33.3	0	0	0	0	3	1.33
**Visual doorbell** The device signals both acoustically and visually the ringing at the door and is mounted in the socket.	93.3	6.7	0	0	0	0	15	**1.07**
**Door detector** Activates light at door opening.	75.0	25.0	0	0	0	0	8	1.25
**Automatic switch** Activates/deactivates electrical devices and automatic plug adaptors from any point in the apartment.	50.0	50.0	0	0	0	0	8	1.50
**Inactivity detector** Noticeably long periods of inactivity (possible indication of a fall) are automatically reported to relatives or an emergency call center.	50.0	50.0	0	0	0	0	4	1.50
**Fall detection bath + toilet** A fall in the bathroom or at the toilet is automatically detected and reported to relatives or an emergency call center.	72.7	27.3	0	0	0	0	11	1.27
**All-off control** Selected electrical appliances or lights are turned off when leaving the apartment by an all-off switch at the door.	83.3	16.7	0	0	0	0	6	1.17
**Home emergency call** Alarms, e.g. in the event of falls, are sent directly to a home emergency call provider.	100.0	0	0	0	0	0	5	**1.00**
**Heating control** The sensor system enables a certain heating temperature to be set and maintained.	33.3	66.7	0	0	0	0	3	1.67
**Installed conventional mobility supporting aids:**								
**Service socket** By pressing lightly on the rotary lever, fixed elbow plugs can be pulled out without any effort.	57.1	35.7	7.1	0	0	0	14	**1.50**
**Object socket** The object socket reduces the risk of accidents. If you trip over a cable or the line of the vacuum cleaner, it releases the plug more easily.	33.3	66.7	0	0	0	0	6	1.67
**Handles bathroom** Handles for easy standing up and sitting down in the bathtub.	58.3	33.3	8.3	0	0	0	24	**1.50**
**Handles toilet** Handles for easy standing up from and sitting down at the toilet.	40.0	40.0	20.0	0	0	0	10	1.80
**Handles balcony** Handles for easy standing up and sitting down on the balcony.	100.0	0	0	0	0	0	8	**1.00**
**Handles corridor** Handles for moving securely through the corridor.	66.7	0	33.3	0	0	0	3	1.67
**Balcony exit** A ramp is installed from the apartment to the balcony to allow barrier-free entry and exit.	81.8	0	9.1	9.1	0	0	11	**1.45**
**Balcony elevation** The base of the balcony is raised to avoid a step between apartment and balcony.	69.2	23.1	0	0	0	7.7	13	1.62
**Overall satisfaction**	63.0	33.3	3.7	0	0	0	27	1.41

### Data analysis

Data were screened prior to analysis for any anomalies (e.g., missing data, outliers, and nonnormality). Data were analyzed using SPSS Statistics for Windows, Version 24 (Statistical Package for the Social Sciences, Chicago, Illinois). Data analysis was primarily descriptive. Scales used were interpreted pseudo metrically resulting in an analysis with mean values (standard deviations), since the use of medians did not allow for sufficient discrimination. Statistical significance of the contingency table (Table 2) was tested by Pearson’s chi-square test.

The cost-benefit analysis focused on quality, price and performance. Price performance ratio and the quality of the installed solutions – smart home solutions and conventional mobility supporting aids –were analyzed and evaluated accordingly. In determining the best price performance, the price of the installed solutions was compared to tenants’ evaluation of the installed solution, while quality performance was determined exclusively on the basis of tenants’ evaluation. Prices of the smart home solutions and conventional mobility supporting aids are displayed separately.

The price and quality assessment of the installed solutions (Table 4) was based on the individual prices for each solution, as given by the manufacturer, in combination with the perceived utility evaluation (quality) with grades ranging from 1 (very good) to 6 (unsatisfactory) as rated by the individual tenants. In a first step, ratings on the conventional mobility supporting aids were looked at separately from the smart home solutions, and in a second step, they were compared with each other.

**Table 4:** Price- and quality-performance of smart home solutions and conventional mobility supporting aids

Installed solution (smart home solutions and conventional mobility supporting aids)	Number of tenants with correspondingly installed solution	Scale (1–6)	Costs in Euro
**Home emergency call**^a^	5	1.00	176
**Handles balcony**^b^	8	1.00	60
**Visual doorbell**^a^	15	1.07	217
**Orientation light**^a^	20	1.15	248
**All-off control**^a^	6	1.17	1001
**Stove safety**^a^	19	1.21	473
**Door detector**^a^	8	1.25	843
**Fall detection bath + toilet**^a^	11	1.27	1389
**LED strip (corridor)**^a^	3	1.33	207
**Balcony exit**^b^	11	1.45	136
**Automatic switch**^a^	8	1.50	192
**Inactivity detector**^a^	4	1.50	1230
**Service socket**^b^	14	1.50	36
**Handles bathroom**^b^	24	1.50	135
**Lighting control**^a^	13	1.54	2630
**Balcony elevation**^b^	13	1.62	125
**Heating control**^a^	3	1.67	1651
**Object socket**^b^	6	1.67	36
**Handles corridor**^b^	3	1.67	135
**Handles toilet**^b^	10	1.80	135
**Tablet**^a^	18	1.83	214

Legend: ^a^ smart home solutions, ^b^ conventional mobility supporting aids

## Results

### Sample characteristics

The response rate was above average in both groups surveyed. A total of 37 of the 46 tenants with installed smart home solutions and/or conventional mobility supporting aids submitted the completed questionnaire, which corresponds to a response rate of 80.4%. In the control group, 64 questionnaires were returned from a total of 100 questionnaires sent out, which corresponds to a response rate of 64%.

The *first part* of the questionnaire was used to collect general personal data. Almost three quarters (74.3%) were between 65 and 80 years old, 25.7% were over 80 years old. Among the tenants in the control group, 63 persons indicated their age, 58.7% of whom were between 65 and 80 years old and 41.3% over 80 years old. In both groups, more than three quarters of the respondents were female. With regard to their housing situation, 42.9% of the tenants with installed smart home solutions stated that they were living alone, which was true for a significantly larger proportion of tenants in the control group, namely 61.7% in total. 43.8% of the tenants in the intervention group were in need of care. In the control group, on the other hand, this was true for a significantly lower proportion, namely 13.6% of the tenants surveyed (Table 1). Of the 37 tenants in the intervention group, a total of 15 persons (40.5%) were in need of long-term care, with care degrees 1 and 2 (care degrees (Pflegegrade) in Germany range from 1 = minor impairment of independence or skills to 5 = severe impairments of independence or abilities that are associated with special demands on nursing care) being the most frequently represented with a share of 33.3% each. None of the tenants in this group had the highest degree of care (care degree 5). Among the tenants from the control group, 8 out of 64 people surveyed required nursing care, which corresponded to 12.5% of the residents. Care degree 2 was most frequently represented (50%). Care degrees 4 and 5 did not occur in this group. The most frequent physical complaints were mobility limitations, both in the intervention group (59.5%) and in the control group (43.8%).

### Residents’ evaluations of smart home solutions

The *second part* of the questionnaire was intended to provide insights into various aspects related to smart home solutions. With regard to the aspects of *care, communication/social contacts* and *safety* in connection with smart home solutions, tenants with installed smart home solutions evaluated the given statements in the questionnaire similarly to the control group (mean values ranging from 1.08–3.73 and 1.3–3.33). Significant results were available for aspects of *communication/social contacts* and *safety.* For example, the survey showed that both groups were fully satisfied that they had regular contact with a reference person in order to report that they were doing well (Table 2 mean 1.08; *n* = 37 and mean 1.70; *n* = 63). In addition, the participants in the study stated that they felt safer in their homes thanks to smart home solutions. This was fully true for tenants from the intervention group (mean 1.55; *n* = 31), and nearly equally true for tenants from the control group (mean 2.38; *n* = 26). Finally, the participants of both groups said that it was fully true for them to know whom to inform immediately in the case of an emergency (mean 1.09; *n* = 35 and mean 1.34; *n* = 61).

### Tenants’ satisfaction with installed smart home solutions and conventional mobility supporting aids

Table 3 shows the mean values of *user satisfaction among tenants with installed smart home solutions,* displaying the three best rated technical and conventional solutions. On a scale of 1 (very good) to 6 (unsatisfactory), the tenants’ assessments of the smart home solutions and conventional mobility supporting aids installed in their homes were in the range from very good to good. The technical solutions were rated with average mean values between 1.00 and 1.83, while the ratings for conventional mobility supporting aids ranged between mean values from 1.00 to 1.80.

The highest rated component of the installed smart home solutions was the “home emergency call” (mean 1.00), which for example in the event of falling, forwards an emergency call to the corresponding emergency call centre. The “visual doorbell”, which signals the ringing of the doorbell to tenants with impaired hearing both acoustically and visually by means of a wireless bell, was rated second best (mean 1.07). In third place was the “orientation light” (mean 1.15), which activates a glare-free floor lighting at night when leaving the bed. The “tablet”, which is used to operate the automated modules, such as the “light control”, received the lowest rates (mean 1.83). Regarding the installed conventional mobility supporting aids, the item “handles balcony” was rated “very good” (mean 1.00), followed by the “balcony exit/elevation” (mean 1.45) and the components “service socket” and “handles bathroom” (mean 1.50). The lowest rating was given to the “handles toilet” (mean 1.80). Overall, the tenants’ satisfaction with all installed smart home solutions and conventional mobility supporting aids was rated “very good” (mean 1.41).

### Cost-benefit analysis of the installed smart home solutions

#### Price performance

With regard to price performance, the best result among the conventional mobility supporting aids (Table 4) was achieved by “handles balcony” (grade 1.0 = “very good”), which in addition had low acquisition costs. Comparable results were also achieved for the “balcony exit/elevation” (grade 1.45 = “very good”) as well as for the “handles bathroom” and the “service socket” (each grade 1.5 = “very good”), of which the service socket was at a lower price. None of the installed conventional mobility supporting aids was rated below 1.8 (grade 2 = “good”) or had acquisition costs (including installation) of more than 140 euros. Among the smart home solutions, the “home emergency call” and the “visual doorbell” achieved best price performances with a rating of 1.0 (“very good”) and 1.07 (“very good”) respectively. Comparable results were achieved for “orientation light” (1.15), “all-off control” (1.17), “stove safety” (1.21), “door detector” (1.25) and “fall detection” (1.27). However, a closer look at the acquisition costs shows that the “home emergency call” (176 euros), the “visual doorbell” (217 euros) and the “orientation light” (248 euros) are the most cost-effective. In contrast, the other systems rated “very good” were considerably more expensive at prices of over 1000 euros. The most expensive system to install was the “light control” with 2360 euros. In terms of grading, none of the smart home solutions was rated below 1.8.

#### Quality performance

Among the conventional mobility supporting aids, the “handles balcony” achieved the best grade of 1.0 (“very good”), while the “balcony exit/elevation” was rated at a similarly high quality (grade 1.45 = “very good”). All ratings ranged from 1.0 to 1.8, meaning that all installed conventional mobility supporting aids were rated between “very good” and “good”. Best quality performance among the smart home solutions was achieved by the “home emergency call” with a grade of 1.0 (“very good”), followed by the “visual doorbell” (grade 1.07 = “very good”) and the “orientation light” (grade 1.15 = “very good”). However, with one exception in each group (“tablet” – grade 1.83; “handles balcony” – grade 1.0), the smart home solutions were overall rated better than the conventional mobility supporting aids (1.0 to 1.5 vs. 4.45 to 1.8).

In summary, all conventional mobility supporting aids were rated at 1.53; all smart solutions at 1.34 on average.

## Discussion

With regard to the research questions to be examined focusing on usability, user satisfaction and price-performance ratio of the installed smart home solutions and the conventional mobility supporting aids, it can be concluded that the installation of the corresponding solutions was positively evaluated by the tenants (overall rating 1.41).

In general, the installed smart home solutions were rated better by the users than conventional mobility supporting aids. At this point, however, it should be pointed out that the poorer evaluation of the installed conventional mobility supporting aids was partly due to unfavourable structural conditions for an optimal installation of the devices. For example, bathrooms were too narrow to adequately install grab handles, and balcony elevations for easy entry and exit, which had actually been evaluated and carried out positively, created a difference in altitude between the apartment and the balcony, which had to be adjusted afterwards.

Analysis of the price-performance ratio has shown that the installed smart home solutions are basically more cost-intensive than conventional mobility supporting aids. However, in contrast to the less expensive built-in conventional mobility supporting aids, it must be taken into account that, although smart home solutions are more expensive, they also contribute significantly to an increased security for the tenants. Inactivity detectors and fall detections can, for example, be linked to different terminal devices, and the notification of the home emergency call may directly be connected to a home emergency call provider. If an emergency call is not available, the information on the event can be transmitted to the mobile devices of the caregiving relatives. In view of the results with an average grade of 1.83 for the tablet and 1.54 for the lighting control, it must be taken into account that tablet training courses were being coordinated at the time of the survey and that problems with lighting control were currently being dealt with by the manufacturer. In the case of lighting control, there were occasional difficulties with the automatic switch.

Not all of the products classified at high quality by the tenants were expensive. Installed solutions up to 250 euros included products that were rated “very good” by the tenants and can therefore be recommended both, from a nursing science and health economics point of view. These include in particular the “home emergency call”, the “visual doorbell” and the “orientation light”.

Overall, it is noticeable that all technologies improve communication possibilities and increase the feeling of security. Visitors are less often “overheard” by the visual doorbell, an emergency call system contributes to the sense of security from the user’s point of view, and orientation lighting offers tenants the opportunity to move around safely, even in the dark and thus, prevent falls. Another study points to the benefits of technical innovations in the home setting [20]. These assessments are also reflected in the comparative survey between the 37 tenants with built-in solutions and the control group. The 37 tenants tended to show a better evaluation of communication and autonomy aspects in their newly created living environment. In some aspects – despite the small sample size – statistically significant differences were found, for example with regard to the statement that the smart home solutions enable a greater sense of security in the apartment.

In addition to the communication possibilities and the feeling of security, another important aspect is the user-friendly handling (usability) of smart home solutions. A prior examination of the usability and user-friendliness (benefit estimation) is an essential prerequisite for the purchase and subsequent integration of smart home solutions in the households of the users [21]. The assumption that older adults generally have a low level of acceptance of technology cannot be supported. Although there is general uncertainty about the new technical devices, this is due to little or no previous technical experience [22–24]. Since almost all users (96.3%) rated the smart home solutions as positive, a negative attitude of older adults towards new technologies can be largely ruled out. Tenants in the intervention group with installed smart home solutions and tenants in the control group without any installed solutions evaluated the actual (intervention group) and the potential (control group) use of smart home solutions in a positive way with similar results. This may indicate a largely unbiased attitude with regard to technical possibilities for supporting security in older adults’ own homes. However, trainings could contribute to reduce possible existing uncertainties among users.

Moreover, a focus must be placed on the financial implementation or the assumption of costs for the acquisition and installation of smart home solutions. With regard to the German legislator’s target of “outpatient rather than inpatient”, it is essential to create conditions to enable elderly people (in need of care) to remain in their own homes as long as possible. Higher additional payments by the health insurance funds for remedies and aids are a first step in this direction. Current questions regarding whether there should be a cost sharing by the users and, if so, how much, or whether selected smart home solutions can be included in the catalogue of assistive devices for services financed by the health insurance funds should be evaluated in the near future [2]. At the same time, the possibility of integration in households should also be examined in advance, since not all households may have connections or suitable structural conditions, and configurations may interfere with other technologies [25]. These are currently important and necessary developments, but this is not enough: with regard to demographic changes and the fact that more and more old and very old people want and “should” continue to live in their own homes, innovative solutions to improve their situation must be considered. In the future, solutions will be needed to ensure the safety of the (care-dependent) residents, which will enable them to live in their own homes until old age. Smart home solutions allow early detection and elimination of dangers, for example to avoid falls or emergencies or to be able to react appropriately and immediately in emergencies [26, 27].

A further point to be considered is the safety of the newly installed smart home solutions. Failure risks or defects of the systems should be checked precisely in advance, so that suitable measures can be taken, if necessary. This means that the installed systems must be suitable for everyday use. Another important point is to ensure accessibility in order to guarantee prevention of possible care phenomena and care problems. In the future, it can be assumed that the possibilities of smart home solutions, especially with regard to networking, will be expanded, so that privacy and data protection will become increasingly important [28, 29].

From the information available, it can be concluded that, in view of the ongoing demographic changes and the associated social changes towards a more dynamic lifestyle for older adults, a special focus should be placed on the further development of smart home solutions [30, 31].

With regard to the initial research questions in this study, it can be stated that tenants report an effective benefit in the installed smart home solutions. In addition, the study showed that smart home solutions generally have a benefit for tenants in terms of the price-performance ratio, even taking into account the more cost-intensive smart home solutions. Finally, the technical solutions were predominantly rated “very good” in terms of the price-performance ratio.

Overall, smart home solutions were rated slightly better than conventional solutions, although the significance threshold was only just missed. This can also be understood as an indication for a high degree of willingness to use technical solutions among elderly and old adults.

### Limitations

Three limiting methodological problems have to be considered in the study carried out: First, the small sample size was small, which is due to the fact that smart home solutions were only installed in 27 apartments of the respective housing association. Second, the actual testing phase of the installed smart home solutions and conventional mobility supporting aids by the users was quite short due to the fact that the installation of some of the corresponding solutions was completed four weeks before the start of the survey. A third methodological problem is, as it is often the case in surveys, the social desirability of the response behaviour. Social desirability is promoted, among other things, by the adaptation to structural characteristics, in this case the installation of smart home solutions and conventional mobility supporting aids by the own housing association. However, socially desirable behaviour may also be promoted by the design of the survey instrument. In order to prevent this tendency as far as possible, emphasis was placed on specific questions oriented towards the individual installed solution or aid in order to reduce the willingness and also the possibilities for criticism. An additional methodological precaution against socially desirable response behaviour was the accompanying letter, in which the tenants were informed that they would participate in the survey voluntarily and anonymously.

Moreover, regarding statistical significance, multiple testing should always be considered. However, since the number of chi-square tests in our study is limited, we assumed the type 1 error to be rather small and thus, further statistical tests have not been carried out.

## Conclusions

The analysis of the study results leads to the conclusion that smart home solutions are a useful supplement to enable elderly adults and/or people in need of care to remain in their own homes as long and safely as possible. Modularized offers consisting of different components of smart home solutions might be recommendable, as costs for the installation can be reduced significantly and an individual composition according to the requirements is possible. In order to best meet the German legislator’s target of “outpatient before inpatient”, a stronger consideration of technical solutions in the list of aids by the National Association of Statutory Health Insurance Funds (German GKV-Spitzenverband) should be considered in the near future. At the same time, corresponding technical solutions can support the approach of the German Care Strengthening Act III (Pflegestärkungsgesetz III), which came into force on 1 January 2017 and which, among other things, aims to develop social rooms in such a way that people in need of care can live in their familiar home and family environment for as long as possible.

## Supplementary Information

**Additional file 1.** Smart@Home_Questionnaire English version. Translation in English language form the original questionnaire in German language.

## Data Availability

The datasets used and/or analyzed during the current study are available from the corresponding author on reasonable request.
