# Supplementary material for: Smart@home – supporting safety and mobility of elderly and care dependent people in their own homes through the use of technical assistance systems and conventional mobility supporting tools: a cross-sectional survey
Source: BMC Geriatr. 2021 Mar 24;21:205. doi: 10.1186/s12877-021-02118-9 (PMC7992959; doi:10.1186/s12877-021-02118-9)
Supplement: Supplementary file 1 — Additional file 1. Smart@Home_Questionnaire English version. Translation in English language form the original questionnaire in German language. [file 12877_2021_2118_MOESM1_ESM.pdf]

Coding: ☐☐

Personal data

Age in years: 65-70 ☐ 71-75 ☐ 76-80 ☐ 81-85 ☐ 86-90 >90 ☐

Sex: male ☐ female ☐

Living alone: yes ☐ no ☐

Care degree: yes ☐ no ☐

if yes, which care degree: 1☐ 2☐ 3☐ 4☐ 5☐

Do you have any physical ailments or problems?

Chronic pain ☐ Mobility limitations ☐ Urinary incontinence ☐

Other ailments: ________________________________

Smart home solutions from GESOBAU were installed in my apartment: yes ☐ no ☐

Smart home solutions from other providers were installed in my apartment: yes ☐ no ☐

**Please indicate to which extent you agree or do not agree to the following statements.**	**fully agree**	**rather agree**	**partly agree**	**rather not agree**	**not agree**
I would like to live in my apartment even if the need for care arises.	o	o	o	o	o
Even with increasing need for care (up to being bedridden), I want to live in my apartment.	o	o	o	o	o
Smart home solutions (e.g. orientation light on the ground, fall detection sensor) enable me to live longer in my own home.	o	o	o	o	o
I have a person with whom I am in regular contact to report that I am doing well.	o	o	o	o	o
I know whom I can inform immediately in the case of an emergency.	o	o	o	o	o
I leave my apartment regularly to maintain social contacts.	o	o	o	o	o
I feel safe in my apartment.	o	o	o	o	o
The installed smart home solutions make me feel safer in my home.	o	o	o	o	o
Smart home solutions in my home lead to more security.	o	o	o	o	o
I can move independently in my apartment.	o	o	o	o	o
I can orientate myself well in my apartment even in the dark.	o	o	o	o	o
I am afraid of forgetting to switch off electronic devices and causing fire damage.	o	o	o	o	o
I am afraid of forgetting to switch off electronic devices and causing water damage.	o	o	o	o	o
I feel comfortable in my apartment.	o	o	o	o	o

**Please tick whether the respective smart home solution is available to you in your home.**	**Please rate the smart home solutions installed in your home using a grading scale from 1 (very good) to 6 (insufficient).**					
**INSTALLED SMART HOME SOLUTIONS**						
	**1**	**2**	**3**	**4**	**5**	**6**
Tablet no ☐ yes ☐						
Stove safety no ☐ yes ☐						
Orientation light no ☐ yes ☐						
Lighting control no ☐ yes ☐						
LED strip (corridor) no ☐ yes ☐						
Visual doorbell no ☐ yes ☐						
Door detector no ☐ yes ☐						
Automatic switch no ☐ yes ☐						
Inactivity detector no ☐ yes ☐						
Fall detection bath + toilet no ☐ yes ☐						
All-off control no ☐ yes ☐						
Home emergency call no ☐ yes ☐						
Heating control no ☐ yes ☐						
**INSTALLED CONVENTIONAL MOBILITY SUPPORTING AIDS**						
Service socket no ☐ yes ☐						
Object socket no ☐ yes ☐						
Handles bathroom no ☐ yes ☐						
Handles toilet no ☐ yes ☐						
Handles balcony no ☐ yes ☐						
Handles corridor no ☐ yes ☐						
Balcony exit no ☐ yes ☐						
Balcony elevation no ☐ yes ☐						

How satisfied are you overall with the aids and smart home solutions installed in your home? **1** **2** **3** **4** **5** **6**

Thank you for your participation!
